# A Privacy Preservation Model for Health-Related Social Networking Sites

**DOI:** 10.2196/jmir.3973

**Published:** 2015-07-08

**Authors:** Jingquan Li

**Affiliations:** ^1^ Health Informatics Research College of Business Texas A&M University-San Antonio San Antonio, TX United States

**Keywords:** social networks, privacy, security, threat modeling, privacy preservation model, electronic health records, health care

## Abstract

The increasing use of social networking sites (SNS) in health care has resulted in a growing number of individuals posting personal health information online. These sites may disclose users' health information to many different individuals and organizations and mine it for a variety of commercial and research purposes, yet the revelation of personal health information to unauthorized individuals or entities brings a concomitant concern of greater risk for loss of privacy among users. Many users join multiple social networks for different purposes and enter personal and other specific information covering social, professional, and health domains into other websites. Integration of multiple online and real social networks makes the users vulnerable to unintentional and intentional security threats and misuse. This paper analyzes the privacy and security characteristics of leading health-related SNS. It presents a threat model and identifies the most important threats to users and SNS providers. Building on threat analysis and modeling, this paper presents a privacy preservation model that incorporates individual self-protection and privacy-by-design approaches and uses the model to develop principles and countermeasures to protect user privacy. This study paves the way for analysis and design of privacy-preserving mechanisms on health-related SNS.

## Introduction

Health-related social networking sites (SNS) are websites that enable the connection of users and facilitate the exchange of health knowledge and information. Physicians can connect with their peers and collaborate on patient cases and other medical topics to improve health care delivery and patient outcomes at sites like Doximity. Patients with life-changing illnesses can find other patients like them, discuss and track medical conditions, and give and receive support at PatientsLikeMe. Before the advent of SNS, medical providers and pharmaceutical companies spread the word to encourage participation in wellness and disease management programs. Today, websites such as Inspire and DailyStrength provide users with the opportunity to share information and stories about healthy living, thereby supporting and inspiring others. The proliferation of these sites is building a new health-information technology business prophesied on the belief that the wisdom of crowds really is smarter than any one person, no matter how well researched the individual person.

However, users reveal vast amounts of personal health information on a health-related SNS. They may also join other social networks or websites and enter personal information and other specific information covering social, professional, and health domains into other websites. There are many possible ways that users’ privacy can be compromised: data misuses, disclosures to intruders, accidental data releases, disclosures to third parties and apps, and user profiling across multiple social networks. A recent incident in which a major media monitoring firm improperly scraped personal data from PatientsLikeMe demonstrates significant privacy risks for online health information [1]. In the United States, health care providers disclose patient information without patient authorization in violation of the Health Insurance Portability and Accountability Act (HIPAA), the Health Information Technology for Economic and Clinical Health (HITECH) Act, and/or state privacy laws and can be subject to fines and other penalties. In the age of Facebook and Twitter, however, many patients themselves volunteer to post their personally identifiable information (PII) and sensitive health information on multiple social networks.

While privacy concerns in social networks are well recognized by prior research [2-7], the literature on innovative privacy-preserving models and technical standards is quite limited. Based on expert opinions on the major privacy concerns, the effectiveness of possible solutions, and the requirements for developing privacy-preserving social network apps, Weiss [7] proposed a privacy threat model for data portability in social network apps. However, this work concentrated on privacy in the sense of visibility and transparence, that is, transparent and open privacy handling practices, and not so much on the privacy-preserving mechanisms that need to be developed. To address the privacy issues caused by the central SNS provider, such as data misuse and leakage risk, it has been proposed to decentralize social networking services [8-10]. However, to our best knowledge, current health-related SNS are predominantly logically centralized services and the underlying business model relies on access to the user-generated content, resulting in the impracticality of the decentralized SNS approach. There is a strong need to develop a privacy model that can protect user privacy in the complex social networking environment. This paper addresses this gap by identifying the most important threats to users and SNS providers and proposes a privacy preservation model to address the privacy challenges of health-related SNS. This paper first analyzes privacy concerns related to health-related SNS. It then develops a threat model and articulates some principal threats. Since current privacy solutions such as end-user license agreements and privacy settings are inadequate to address the threats, this paper presents a privacy preservation model that integrates both individual self-protection and privacy-by-design approaches and uses the model to develop principles and countermeasures to protect privacy.

## Privacy Concerns Related to Social Networking Sites

To illustrate privacy issues with health-related SNS, we analyze the Inspire platform. Inspire may be considered an illustrative case of patient sites that offer a privacy policy and settings to address users’ privacy concerns.

Inspire is an online health and wellness support community for patients and caregivers. Inspire is provided by ClinicaHealth, Inc., and is composed of more than 190 disease-specific communities. As of December 2014, Inspire has over 400,000 registered members and 700,000 unique visitors each month.

Inspire is free for individuals and non-profit patient advocacy associations [11]. Its business model largely depends on advertising revenue and partnerships with many third-party companies [12]. Inspire helps companies and researchers find likely clinical trial participants. Clinical trial sponsors pay Inspire for this service. Inspire also offers health-focused research services to commercial companies. User-generated content has high value for the companies to conduct secondary research and issues analysis. Furthermore, Inspire makes money from selling targeted advertising.

The products and services of Inspire are essentially user profiles and user-generated content. Inspire collects and stores three types of information from users: personal profile, user-generated content, and Web behavior information. At its registration page, Inspire asks a new user to provide certain personal information, including a functioning email address, postal code, gender, date of birth, user ID, and password. A user is also given an option to provide additional personal information to create an extended online profile [13]. User-generated content is all the information a user posts on the site or communicates with other users, including disease conditions, treatments, family history, and possibly personal information generated by the user. Web behavior information is information on how a user uses different features of the site collected through cookies. Inspire may combine this information with the profile [13].

Inspire strives to create a secure environment where users connect with each other around shared conditions and share relevant information about their health and the health of their loved ones. When people’s personal information is involved, however, there are several privacy concerns. First, Inspire may reveal personal information to other users and outsiders. When a user registers at the site, the profile becomes visible to other users of the site and the profile may also be found by visitors of the site using Inspire’s search functions. Although users can use the privacy settings to control access to their profiles, they may not have the knowledge and technical skills to understand the settings and change their own settings appropriately.

Second, Inspire has the right to use personal information for various purposes without user control. For example, it may use personal information to present targeted content, including advertising or requests either from ClinicaHealth or from a third party. Users have no control over the collection and use of personal information by Inspire and its affiliates. Inspire makes clear under its privacy policy: “ClinicaHealth may share your email address and profile information with the organizations that sponsor Inspire groups that you join” [13]*.*

Third, although Inspire does not disclose a user’s PII to third parties without consent, it may share health information with third parties on an aggregate or other basis that does not disclose user identity or contain PII [13]. However, concerns have been raised about the sufficiency of popular de-identification methodologies such as merely stripping names and addresses; data mining tools make it possible to reverse-engineer PII from weakly de-identified user information [1]. Furthermore, user-generated content, which may contain PII accidentally revealed by users, is open to the community, outsiders, and third parties.

Fourth, Inspire is an open community. Anyone with a valid email address can sign up for Inspire and then view the content on the site. This raises the problem of unauthorized access by unintended users. Inspire is also vulnerable to attacks from malicious intruders, such as data scraping and social engineering attacks.

**Table 1:** Examples of health-related social networks and general social networks.

Social network	Description	Privacy practices
CarePages.com	CarePages is a community of people collaborating to share the challenges, hopes, and victories of anyone facing a life-altering health event.	Privacy settings include “Community”, “Friends & Family”, and “Invitation Only”; secondary use of personal information; CarePages combines personal information with the data received from third parties to target advertising [14].
CureTogether.com	CureTogether provides a service whereby patients and researchers come together to share information and find cures for chronic diseases.	Privacy settings include “Public”, “Research”, “Friends”, and “Private”; secondary use of personal information; disclosing de-identified information to third-party researchers [15].
DailyStrength.org	DailyStrength is a health network of people sharing advice, treatment experiences, and support.	Users and visitors can see any information users provide; secondary use of personal information; DailyStrength reserves the right to use and disclose de-identified information to third parties at its discretion [16].
Inspire.com	Inspire has mini social networks for different diseases and health conditions, each sponsored by health organizations.	Privacy settings include “Public”, “Members”, “Friends”, and “Private”; secondary use and disclosure by the SNS provider and its affiliates; sharing aggregate personal and health information with third parties [13].
PatientsLikeMe.com	PatientsLikeMe is a social network that enables people to share health experiences that can improve the lives of patients diagnosed with chronic diseases.	PatientsLikeMe provides two privacy levels “Public” and “Members”; secondary use of personal information; disclosing shared data to partners and other third parties for use in scientific research and market research [17].
Facebook.com	Facebook is a social network that enables users to create profiles, upload photos and videos, send messages, and communicate with friends, family, and colleagues.	Privacy settings include “Public”, “Friends”, “Only Me”, “Custom”, and “Close Friends”; secondary use of personal information; sharing non-personally identifiable information with advertising and analytics services and disclosing all information to other third parties [18].
Twitter.com	Twitter is a microblogging platform that enables users to send and read short 140-character messages called “tweets”.	Tweets can be “Public” or “Private”; a public user profile, login verification, and tweet location can be configured; secondary use of personal information; sharing personal information with its service providers and third parties [19].

These concerns are also intrinsic to other health-related SNS and general social networks such as Facebook and Twitter. The purpose and privacy characteristics of top patient sites and general social networks are summarized in Table 1 [14-19]. Furthermore, the security characteristics of these sites are vague. Without effective privacy controls, health-related SNS may disclose the information not only to their business partners but also to unintended individuals and entities. The concern is not just about data mining and marketing that could influence patients to seek drugs they do not need or to spend more money on branded drugs rather than generics. More broadly, employers, health insurers, and/or identity thieves could gain access to users’ profiles, leading to negative consequences, including privacy compromise, social embarrassment, discriminations from employers and insurance companies, identity theft, and so forth [5,20]. Because health-related SNS are not HIPAA-covered entities, these concerns are very real and must be addressed seriously. When users lose their trust and confidence in the ability of a health-related SNS to protect privacy, that company’s reputation will be irreparably damaged.

## A Threat Model

For users, a health-related SNS consists of a set of users, a set of mechanisms for exchanging information, a set of binary relations between users, a set of search functions, and a site operator.

**Figure 1:**
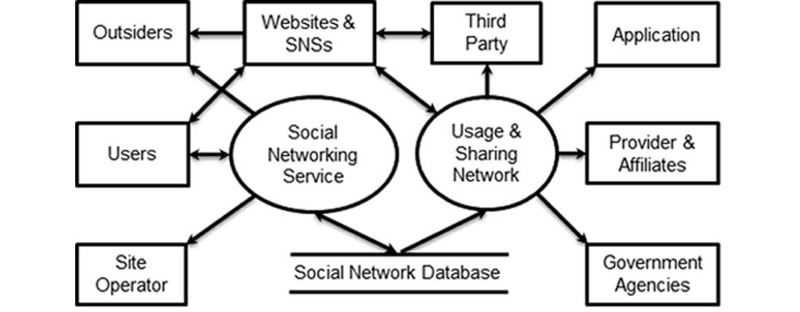
A health-related social networking site.

The SNS provider and its affiliates may use health information for many purposes. It may also release health information to various third parties and apps or enable exchange of information with other social networks. We may include these additional actors into a usage and sharing network that involves the SNS provider and its affiliates, a set of third parties that collect data from the site, a set of apps that users may invoke within the site, a set of other SNS or websites, and government agencies, including law enforcement and public health. An information flow diagram for a health-related SNS is shown in Figure 1.

In the social network, a user creates a personal profile, content, and connection network on a health-related SNS. The user may also join other social networks in order to enjoy different social networking services and enter personal health information into other SNS or websites. The site and other websites permanently store the information into their own databases. The site operator uses the information to control the site. The site may make the information visible to other users or even to unintended outsiders including visitors, fake accounts, and attackers. Outsiders may also draw information from other websites.

In the usage and sharing network, the SNS provider and its affiliates may use the accumulated health information for commercial purposes. They may disclose the information to third parties (eg, researchers, marketers, insurers, employers) that may also collect information from other social networks that users have joined and show some information collected from the current site on other websites. The SNS provider may also permit users to launch various apps that draw information from user profiles in order to create targeted materials. Furthermore, the SNS provider and other SNS providers may share their databases and link different user accounts across multiple SNS due to the collection of more personal health data. Finally, the SNS provider may release the information to government entities for law enforcement or social uses.

## Privacy Threats

### Overview

The process of identifying threats to users should recognize users’ interest in protecting personal information from parties with which they do not consent or intend to share it. Users are also concerned that personal data may be used in the wrong way or for the wrong purpose. Thus, we look at four key elements in defining a privacy threat—the actors who disclose information, the actors who receive information, the types of information involved, and the purpose. We outline the principal threats of SNS below.

### Excessive Revelation of Personal Health Information

Many users have provided unprecedented amounts of detail about their lives, including PII and sensitive health information. Some people hope that exchange of health information will help them access health advice, receive and give social support, manage their conditions, or improve their overall health and quality of life [21,22]. However, health-related SNS may make the information easily accessible to unwanted audiences. Some people may reveal their personal information for the sake of the greater good. Yet they typically have no way of knowing whether their profiles contribute directly to the development of more effective treatments or simply become a lucrative asset for sale. The shared information may contain personal information such as real name and photos, together with their medical conditions. Once personal health information is compromised and the resultant harm is done to that person, it cannot be withdrawn and made private again [20]. Furthermore, users post not just great amounts of private information about themselves but information about other people such as their family members and friends. Although some medical research programs need health information about patients’ relatives, disclosing medical information about other people is considered a privacy violation. Individuals sharing information on health trends can, if their submissions are aggregated, reveal information about the health issues affecting their local communities or ethnic groups [4].

### Access and Use by Other Users and Visitors

Personal health information may become visible to other users, and visitors may also find the information via the website’s search features or even Google searches. This raises the problem of inappropriate access and use by other users and visitors. Even if users can control the access to their own profiles, they may not control what other users and visitors reveal about personal information posted in a public area. For example, other users could be untrustworthy and steal an individual’s health information and use it for their own purposes. They may disclose the information to the person’s employer or insurer or post it on the Internet.

### Secondary Uses and Disclosures by the Social Networking Site Provider and its Affiliates

After users share their personal health information with a health-related SNS, they may lose control over the distribution of their information. The SNS provider has unlimited access to all the information. Ultimately the SNS provider expects that the information will generate insights with considerate scientific as well as economic value. Users are extremely vulnerable because they have little control over the collection, use, and disposition of their information. Privacy can be compromised in many possible ways: targeted advertising, secondary use of the information for research, direct misuse, creation of a permanent record of personal profiles, accidental information release by a site operator, etc.

### User Profiling Across Multiple Social Networking Sites

Many users join multiple social networks for different purposes. This means a user may hold multiple profiles, which are stored and shared in different SNS [23-25]. For example, a user creates an account on Facebook mainly to communicate with friends and families, as well as to share pictures and videos with them. In the meantime, she provides her professional profile and establishes her professional networking on LinkedIn. Furthermore, she stores her personal health records and shares her treatment and symptom on a health-related SNS.

Third parties and companies may use different user accounts and their social relations to connect multiple social networks and produce aggregated user profiles [24,25]. These aggregated user profiles would be immensely valuable to companies looking to market products or services or, in the case of employers, screen potential job applicants. Furthermore, companies can integrate multiple social networks and conduct social network analysis and mining tasks on the integrated social networks [26]. Individual published social network data capture only a partial picture of a user’s complete social network. Integration of multiple online and real social networks provides a more complete picture of a user’s social network.

Unfortunately, such user profiling and social network integration is not necessarily always beneficial. For example, malicious third parties and identity thieves may use their own crawler systems to obtain a user’s private information and friend lists. More seriously, such third parties and individuals could create fake accounts pretending to be this person and then solicit others to connect [25]. These fake accounts can be abused to deliberately leak the user’s private information and friend lists to malicious intruders, which could quickly turn into identity theft and fraud, losing a job, hurting relationships, or even worse.

### Secondary Uses and Disclosures by Third Parties

The SNS provider may disclose personal health information to third parties and apps. Users cannot assess the risks of divulging personal information unless they know the set of organizations to which their information may be disclosed, and the uses to which it may be put [27]. Because health information is of high commercial value, the accessibility and manipulability of the information creates economic pressures for its use and disclosure for a widening range of commercial and industrial uses. The SNS provider may also allow third-party websites and apps to automatically have access to users’ personal information. Data portability technologies may allow many websites and apps to be linked together, letting them share both dynamic content and the nature of the relationships of their users [3,4]. For example, an SNS may communicate with advertising servers, which produce targeted advertising based on details contained in user profiles. The ability to draw data from multiple websites and apps may allow third parties to create a comprehensive digital profile of private data, accumulating more than what a user would have predicted [2,28].

### Inability to Detect Sources of Privacy Violations

A health-related SNS cannot assure users’ privacy if it lacks automated tracing mechanisms to monitor and track uses and potential misuses of personal information. Visibility and transparency has not been a strong point of health-related SNS. Information mash-ups and the combination of apps and multiple different types of SNS [24] create unexpected information flow through “back channels”, impeding users’ ability to get a clear view of the way their data are propagating [5]. Different actors (eg, users and apps), linkages, and roles are having dynamic interactions with each other through different ways across multiple social networks or websites. Thus, it is hard for a user to identify the core elements (eg, bridge, hub, broker, power user, proxy) responsible for information dissemination among multiple SNS and find their implicit and explicit relationships with other SNS [24]. Users are often incapable of defending their privacy just because they do not know that their privacy is even endangered. Privacy policies, especially relating to third parties, apps, and social network data sharing and integration, are often vague, uninformative, and seldom reflect users’ expectations [2,28,29].

### Outsider Attacks

A health-related SNS is vulnerable to attacks from malicious outsiders, such as data scraping and social engineering attacks. Data scraping is a technique that trolls online communities, discussion boards, blogs, and chat rooms looking for personal information that can be used for fraud or any other purposes. For example, data scrapers may choose to work surreptitiously through hidden programs, or they may sign up with a fake email address in order to obtain personal information from unsuspecting users. A patient site also creates a perfect social and ecological environment for spear phishing, viruses, worms, spyware, spoofing, and Web app attacks, facilitated by human vulnerability and easily accessible user profiles [28]. Furthermore, a health-related SNS is vulnerable to social engineering techniques that exploit low entry thresholds to trustful health communities [3].

## A Privacy Preservation Model

### Overview

Health-related SNS have unique needs to address the principal threats to users and SNS providers not only because personal health information is highly sensitive but also because privacy is essential for building trust, which is the foundational currency of health communications. Today, the dominant approach is a combination of end-user license agreements and privacy settings. Privacy by license agreements is problematic because users have to accept these agreements prior to using SNS services even if they are concerned about privacy. Empirical and theoretical research suggests that users often lack enough information to make privacy-sensitive decisions and, even with sufficient information, are likely to trade off privacy for health benefits [30]. Moreover, the terms of these agreements seldom reflect users’ expectations because they can be created and changed only by SNS providers, not by users [29,31].

Current privacy settings provided by most health-related SNS suffer a number of drawbacks. First, since most SNS make “public” their default settings, users may forget to change the default settings. Second, individual self-control is constrained by the user’s awareness and education and by the technical design of an SNS, which may impede easy and effective management of settings regarding the access, use, and disclosure of personal information [2]. Furthermore, privacy settings give users control over who sees what on each profile, but they give users little control over what the SNS provider and its affiliates reveal about them. Therefore, asking individuals to assume full responsibility for policing the use of their profiles by other users and visitors is no longer reasonable, nor does it offer sufficient checks against direct misuse and improper disclosure of personal information by the SNS provider and its affiliates.

**Table 2:** Privacy threats and countermeasures.

Privacy principles	Privacy threats	Countermeasures
Safe, flexible, and user-friendly privacy settings	Excessive revelation of personal health information; improper access and misuse by other users and visitors; secondary uses and disclosures of personal information; user profiling across multiple SNS.	Banning personally identifiable information; flexible and user-friendly way of setting privacy preferences; individual choice and consent; visualization of connection network; integration of privacy and security settings across multiple SNS.
Privacy by design	Secondary uses and disclosures by the SNS provider and its affiliates; secondary uses and disclosures by third parties; user profiling across multiple SNS.	Sharing de-identified data inside or outside an SNS; limiting use, disclosure, and retention; deleting user accounts upon request; a global privacy preservation model for data sharing and integration across multiple SNS.
Privacy audits	Inability to detect sources of privacy violations; user profiling across multiple SNS.	Audit trails; auditing and monitoring; transparency of data-handling practices; options for users to report privacy invasions; auditing usage and data sharing across multiple SNS.
Security for privacy	Outsider and insider attacks.	Technical barriers such as multifactor authentication, encryption, continuous monitoring, and security analytics; organizational measures such as user education and awareness, options for users to report a security incident, and breach notification and enforcement.

Instead, a privacy model based on a shared responsibility between the SNS provider and users may be better suited as a means of effective protection for both the SNS and its users. User profiles, user-generated content, and social links are the most valuable asset for the SNS provider, and it should be in the best interests of the SNS provider to find solutions to protect those assets through effective means. Therefore, this paper assumes that both the SNS provider and users share the same values concerning protection of user privacy. Direct misuse and improper disclosure of personal information in the usage and sharing network (Figure 1) can lead to conflicting interests for users and the SNS provider. The conflicting interests can be resolved by other means (eg, regulations [2], decentralized social network services, and cryptographic solutions [8-10]) that fall beyond the scope of this paper. The threat analysis outlined above indicates that privacy protection should be considered on four fronts: user self-control, privacy-preserving mechanisms, privacy audits, and security mechanisms. Building on early research [2-7,26,31-33] and the concept of privacy by design [34], this paper proposes a privacy preservation model that incorporates both individual self-protection and privacy-by-design principles. Below we identify key privacy principles and countermeasures to address the principal threats of health-related SNS (Table 2).

### Safe, Flexible, and User-Friendly Privacy Settings

Privacy settings play a vital role in matching users’ privacy expectations. Many health-related SNS give options to hide certain types of personal information from other users and visitors through the customization of privacy settings. The SNS provider expects users to choose their privacy settings meticulously using available privacy options. But users’ self-protection behaviors are constrained by their privacy awareness and by the technical design of privacy settings. Safe, flexible, and user-friendly privacy settings allow the user to set privacy preferences easily and effectively. First, a health-related SNS should turn on privacy settings that limit the collection, display, or sharing of PII by default [3]. For example, the SNS would not make any PII publicly viewable until the user takes affirmative steps to allow this. Second, the SNS can provide flexible privacy settings that afford users fine-grained control over each and every piece of personal information so that other users and visitors cannot access it without explicit consent. Privacy could be compromised by the user’s inability to control impressions and manage complex social contexts [7]. It needs to be a major responsibility of the SNS provider to raise the awareness of users and to make its privacy settings very user-friendly. If the SNS enables exchange of information with other SNS or websites, a global model is needed to deal with issues of integration of privacy and security settings across multiple SNS. Third, health-related SNS may provide a means by which users can visualize their current exposure within the community and across multiple social networks. In practice, users have little sense of how their information is accessed and used by other users, visitors, apps, third parties, and other SNS. Graphical displays of the social relations and user accounts linkage across multiple social networks [24,26] would help the user appreciate the potential risks arising from a disclosure and customize their individual settings accordingly.

### Privacy by Design

Privacy by design refers to the philosophy and approach of building privacy into the design and architecture of technologies, business practices, and the underlying technical platforms [34]. The presence of protection for users’ privacy, including data anonymization and purpose limitation, is crucial to gaining the necessary public trust to make the SNS successful. The following privacy-preserving mechanisms have to be taken into account. First of all, the SNS provider may design architectures that apply appropriate privacy-preserving transformations before transferring the information to individuals and entities. There are several transformation techniques. The safe harbor de-identification method attempts to suppress individual identifiers in order to de-identify the data. Health-related SNS might voluntarily comply with the HIPAA privacy rule by deleting 18 common identifiers before disclosure [35,36]. Under the HIPAA privacy rule, data are considered de-identified if the covered entity removed the following identifiers from the data: names, addresses, dates, telephone numbers, fax numbers, email addresses, social security numbers, medical record numbers, health plan beneficiary numbers, account numbers, certificate/license numbers, vehicle identifiers and serial numbers (including license plate numbers), device identifiers and serial numbers, Web Universal Resource Locators (URL), Internet Protocol (IP) addresses, biometric identifiers (including finger and voice prints), full-face photographic images and any comparable images, and any other unique identifying number, characteristic, or code.

An alternative approach, known as statistical anonymization techniques [37-39], desensitizes the data by suppressing quasi-identifiers (eg, postal code, birth date, gender, hometown, and/or other demographics), decreasing precision/accuracy, and/or adding confusion to the information in order to make it more difficult to link de-identified data back to the individual. Properly applied statistical anonymization is an effective tool for protecting privacy and preserving the ability to leverage user-generated content for secondary purposes. Furthermore, health-related SNS may use network data anonymization techniques to reduce the identity inference risks from social network data such as social graph, tagging data, email, or instant messaging. The techniques attempt to suppress the user’s network structure by graph modification approaches and clustering-based approaches [33]. However, the techniques only allow us to investigate the structural properties of a single anonymized social network. In many cases, node identifiers are essential to link data from different social networks. In order to share useful information among different social networks while protecting privacy, Tang and Yang [26] proposed a generalization and probabilistic approach by generalizing social networks to preserve privacy and integrating the probabilistic models for the generalized social network data for social network analysis and mining.

Over the past few years, however, researchers have found that even de-identified data could be re-identified and attributed to specific individuals [40,41]. Third parties and companies are actively seeking end-user information by linking a variety of different data sources and different user accounts across multiple social networks. The more datasets to which third parties and companies have access, the easier such re-identification becomes. Therefore, the SNS provider and third parties should make a public commitment not to re-identify the data for commercial uses without explicit consent and it should contractually prohibit downstream recipients from doing the same. The SNS may also provide privacy-preserving interfaces for third-party apps while still enabling them to deliver customizable content. Current best practices include “privacy by proxy” mechanisms [32].

Second, the SNS provider may limit the collection, use, and disclosure of personal information to the purposes identified in the privacy notice. Personal information shall not be used or disclosed for purposes other than those identified in the privacy notice, except with consent or where required by law. It is a challenge to find a balance between privacy and utility in data sharing and integration across multiple social networks and websites. On one hand, users’ personal information is the most valuable asset for SNS providers and it should also be in their best interest to protect the asset. SNS providers, on the other hand, need to proof their business model by further expanding ways to exploit the value of their users’ personal information. Stringent penalties for misuse and improper disclosure of personal information should be established through federal regulations or contractual mechanisms.

Third, a health-related SNS may provide convenient tools to allow users to destroy their profiles and posts completely, in a timely fashion. These tools should allow users to remove their personal information safely and delete or edit their posts in a user-friendly way.

### Privacy Audits

Privacy audits provide a means of independently verifying that a health-related SNS operates according to its privacy policies. Auditing and monitoring services are not included in the privacy policies of current health-related SNS. A health-related SNS cannot assure users of their privacy and security unless it enables users to request an “audit trail”, detailing when their personal information was accessed, by whom, and for what purpose. A second alternative is to actually audit access and actively notify users in the case of inappropriate access. This principle seeks to assure users that a health-related SNS is operating according to its privacy policies, subject to independent verification. Its component parts and operations are visible and transparent to users. Options for the user to report privacy invasions establish transparency and additional trust in its commitment to adequate treatment of personal information. Furthermore, malicious intruders may use their own crawler systems to obtain a user’s private information and friend lists and infer the user account’s linkage across multiple SNS and websites. It is highly desirable to design a methodology for auditing usage and data sharing and detecting unauthorized access to each user’s personal information across multiple social networks.

### Security for Privacy

Health-related SNS may provide appropriate security safeguards that improve privacy. Intruders are increasingly using complicated techniques via the Internet to steal personal information. Traditional security solutions like firewalls and encryption are no longer the centerpiece against social network attacks. Encryption technology for the transmission and storage of personal information provides enhanced security. But data thieves may steal personal information via fake accounts or launch automated crawling and identity theft attacks across different SNS and obtain a large amount of user private information. Some health-related SNS do not use a validation process during new user’s registration. Weak authentication of registrants through a functional email address, the preferred validation requirement, is not an adequate method and leads to a proliferation of fake accounts populating the network. Therefore, health-related SNS may develop strong multifactor authentication that combines two or more independent categories of credentials: what the user knows (password), what the user has (security token), and what the user is (a biometric characteristic such as a fingerprint). Health-related SNS may also invest in things like continuous monitoring and security analytics solutions that monitor the network 24/7 [42], reporting suspicious activities or vulnerability. Social engineering and phishing attacks are the most important threats to users. Sadly, there is no computer program that can protect the network from social engineering or phishing attacks. The best protection is security education and awareness. Health-related SNS could develop proactive communication techniques that raise the level of education and awareness about dangers of privacy and security breaches. Procedures and policies could also be in place for reporting misuse and illegal activity.

The above-mentioned principles and techniques form the basis of how to address the threats of health-related SNS and other eHealth technologies. In principle, many of the techniques and industry best practices needed to implement and enforce these principles are available, if not deployed on existing health-related SNS. We do not have space to detail all the protections for user privacy in this paper, but only to provide a concise set of countermeasures and to relate the countermeasures to the identified privacy threats (Table 2). Since de-identification and informed consent are key elements of privacy laws, these principles and countermeasures can give a health-related SNS legal cover in case of a privacy breach.

### Conclusions

A health-related SNS benefits from the increasing amount of personal health information willingly shared on its site, but users are likely to be exposed to privacy and security threats. In this paper, we have developed a threat model that highlights the underlying usage and sharing network behind the SNS and shows the principal threats to users. Because the established solutions like license agreements and unsafe privacy settings are inadequate to mitigate the threats, we proposed a conceptual privacy framework that integrates such foundational principles as safe and flexible setting, privacy by design, privacy audits, and security for privacy. The principles and their associated countermeasures provide a practical way to protect privacy against unauthorized individuals or entities. This proposed model can be generalized to other online settings where personal information is available.

Because personal health information is extremely valuable to both the SNS provider and its business partners, there are always economic pressures on the SNS provider to exploit the value of the database it holds—a prospect that becomes even more tempting if the current business model that supports full user control does not generate sufficient revenue. Hence, there is a tension here, because without effective protections, many users would refrain from sharing health information online due to privacy concerns [43], causing the community to fade away. But if the SNS allows users to keep too much of their information private, there will be less content for creating commercial and social value inside or outside the SNS. Consequently, its business will suffer. The main challenge in the future will be to develop privacy-preserving SNS that protect user privacy while still tapping the richness of user-generated content. All involved parties, and at the foremost the SNS developers, need to understand the potential threats that exist and therefore build privacy and security protections into health-related social networks.

## Abbreviations

HIPAA: Health Insurance Portability and Accountability Act
HITECH: Health Information Technology for Economic and Clinical Health Act
IP: Internet protocol
PII: personally identifiable information
SNS: social networking sites
URL: uniform resource locator
